# Spontaneous Coronary Artery Dissection of the Left Main Coronary Artery

**DOI:** 10.7759/cureus.11801

**Published:** 2020-11-30

**Authors:** Avanti Suresh, Amardeep S Parhar, Kerry S Singh, Asseel Al-bayati, Renato Apolito

**Affiliations:** 1 Internal Medicine, Jersey Shore University Medical Center, Neptune City, USA; 2 Internal Medicine, St. George's University, Neptune City, USA; 3 Internal Medicine, University of Baghdad, College of Medicine, Baghdad, IRQ; 4 Cardiology, Jersey Shore University Medical Center, Neptune City, USA

**Keywords:** left main coronary artery, spontaneous coronary artery dissection, scad

## Abstract

Heart attacks in young populations are frequently misdiagnosed as reflux disease or anxiety. Spontaneous coronary artery dissection (SCAD) is a coronary artery disease that classically manifests around the age of 45 years and has a fatal outcome if missed. Since it was first described in 1931, our understanding of SCAD has evolved tremendously, particularly with the advent of advanced coronary angiography. Electrocardiograms can show abnormality in the ST-segment, with an elevation of cardiac damage markers. The involvement of the coronary arteries is variable. The left main coronary artery is frequently reported as the main culprit. We are hereby reporting a case of SCAD in a young woman who presented with a heart attack. Particularly, her age is younger than the reported cases, cardiac catheterization revealed a left main artery dissection with a thrombolytic extension, and her complicated case was managed with coronary artery bypass and heart transplantation.

## Introduction

Spontaneous coronary artery dissection (SCAD) is an infrequent cause of acute coronary syndrome (ACS), which is defined as an epicardial coronary artery dissection that is not associated with atherosclerosis, trauma, or iatrogenic [1]. SCAD is considered a rare disease of the coronary arteries, and it presents majorly as a myocardial infarction (MI) in young women. During SCAD, myocardial damage is caused by the disruption in coronary artery circulation due to the formation of intramural hematoma and/or intimal disruption [1]. Risk factors that predispose patients to SCAD include fibromuscular dysplasia, systemic inflammatory conditions (i.e., systemic lupus erythematosus, rheumatoid arthritis), connective tissue diseases (i.e., Marfans, Ehlers-Danlos), postpartum status, and multiparity [2].

Epidemiologically, SCAD represents 0.1 to 4% of all ACS cases in the U.S [3]. Further analysis demonstrates that nearly 25% of ACS cases in women < 50 years old were caused by SCAD [4]. Classically thought of as a condition affecting mostly young women 43-52 (±10) years, new data reflects increased occurrence in older and postmenopausal women [4, 5]. Overall, the true incidence of SCAD is difficult to gauge, as this disease is very often under and/or misdiagnosed.

Studies have shown that SCAD coexists with certain conditions. Fibromuscular dysplasia is the most common predisposing condition (31.1%) [6]. In a study of 750 patients who presented with acute SCAD, 34 patients (4.5%) were in the peripartum period (from third-trimester pregnancy to within 12 months of delivery) [6, 7]. It has also been found that patients with peripartum SCAD tend to present more acutely and have worse presentation and outcome than those with non-peripartum SCAD patients [7].

Patients with SCAD typically present with ACS symptoms, most classically including chest pain seen in 96% of cases; other symptoms include arm, neck, or jaw pain, nausea/vomiting, and diaphoresis [5]. EKG usually reveals ST-elevation MI (STEMI) or non-ST elevation MI (NSTEMI), with <1% of cases having no elevations in troponins [6]. Most commonly, the left anterior descending (LAD) coronary artery is affected, accounting for 40-70% of documented cases [8]. Many studies have shown that conservative medical strategy has a high success rate compared to percutaneous coronary interventions (PCI). With a conservative medical approach, the dissection was completely resolved and healed, given the patient follows up within 30 days of the diagnosis [6]. PCI is reserved for patients with high-risk features: ongoing myocardial ischemia, left main coronary vessel involvement, cardiogenic shock, ventricular arrhythmia [4]. Moreover, severe cases result in cardiogenic shock requiring mechanical assist devices (i.e., Impella®, left ventricular assist device [LVAD]) and emergent coronary artery bypass graft (CABG), with the most severe cases requiring transplantation [8, 9].

This case report describes a young female without risk factors who presented with severe spontaneous complete left main coronary artery dissection. PCI showed extensive coronary disease that required CABG and advanced cardiac support with Impella placement and extracorporeal membrane oxygenation (ECMO). Eventually, the patient successfully underwent cardiac transplantation.

## Case presentation

A 32-year-old female with a past medical history of asthma presented to the emergency department (ED) with sudden-onset chest pain, which started seven hours prior to presentation. The pain was retrosternal, crushing in nature, and rated seven out of 10 in intensity. The patient reported that the pain radiated to the left arm and back. The patient endorsed concurrent sweating, nausea, and vomiting. On the way to the ED, the patient received four tablets of 81 mg aspirin and 500 ml of intravenous normal saline.

**Figure 1:**
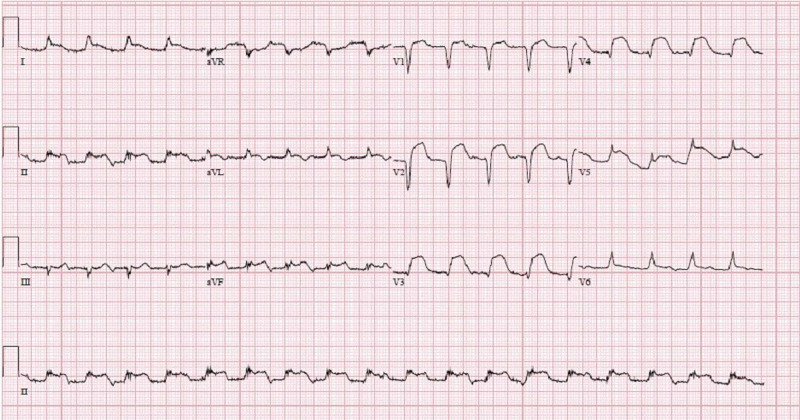
EKG showing >1 mm ST-segment elevations in lead I, II, aVF, and aVL; also >2 mm ST-segment elevations in lead V3-5

In the ED, the patients’ vital signs were measured - the temperature was 97.8° Fahrenheit, blood pressure was 71/39 mm Hg, heart rate was 118 beats per minute, and respiratory rate was 16 breaths per minute. Her oxygen saturation level was 100% on room air. Her complete blood count (CBC) and complete metabolic panel (CMP) on admission were within normal limits except for a glucose of 156 mg/dL, bicarbonate of 18 mmol/L, and an alkaline phosphatase of 30 U/L. An initial electrocardiogram demonstrated >1 mm ST-segment elevations in lead I, II, aVF, and aVL, and >2 mm ST-segment elevations in lead V3-5 (Figure 1). 

The patient was emergently taken for coronary angiography. A coronary angiogram demonstrated dissection of the left main coronary artery extending into the left anterior descending artery, ramus intermedius, and left circumflex artery (LCX) with thrombolysis in myocardial infarction (TIMI) 0 flow (Video 1).

**Video 1:** Coronary angiography showing left main coronary artery dissection

The left ventricle examination revealed mild anterolateral hypokinesia and mildly reduced left ventricular (LV) systolic function. While undergoing catheterization, the patient developed cardiogenic shock and was administered a 400 micrograms bolus of IV phenylephrine followed by infusion according to our institution to achieve a mean arterial pressure >65 mmHg.

Due to the extensive pathological involvement of the patients’ coronary vasculature, the management was deferred to cardiovascular intervention with a salvage coronary artery bypass graft (CABG) operation. Meanwhile, an Impella catheter pump (CP) set at 3.6 L/min flow was inserted to provide circulatory support. While undergoing catheterization, the patient twice developed ventricular fibrillation prompting defibrillation and administration of amiodarone 200 mg IV push. Normal sinus rhythm was ultimately restored, but the patient remained in persistent cardiogenic shock with a mean arterial pressure (MAP) of 40 mm Hg despite Impella P8 flow and maximum vasopressor administration. While awaiting cardiothoracic surgery, the ramus intermedius and LAD arteries were manipulated with coronary wire placement to achieve TIMI III flow distally.

The patient was subsequently intubated and underwent an emergent triple CABG procedure consisting of left internal mammary artery (LIMA) graft to the mid LAD, saphenous vein graft (SVG) to the distal LAD, and SVG to the LCX. During the procedure, the mediastinal extracorporeal membrane oxygenation (ECMO) machine was utilized to provide further circulatory support.

Postoperatively, the patient remained intubated and sedated in cardiogenic shock with an epinephrine drip, Impella CP set to P6, and ECMO at 3200 rpm with 2.3 L/min flow. The decision was ultimately made to transfer the patient to a heart transplant facility where she eventually underwent successful heart transplantation. At the time of publication of this report, the patient reported feeling great without major complications. The patient is on routine follow-up care in the heart transplantation clinic, and she is taking routine immunosuppressants without any symptoms.

## Discussion

Spontaneous coronary artery dissection (SCAD) can be defined as the non-iatrogenic and non-traumatic separation of the coronary arterial wall by intramural hemorrhage, which can occur with or without an inciting intimal tear [10]. It typically affects a young, mostly female population, with patients frequently presenting with an ST-elevation myocardial infarction (STEMI) [11]. The typical age of presentation of SCAD has been reported to be seen primarily in women ≤50 years old, with some studies finding the mean age between 43 and 52 years old [4-6]. While our patient did present with a STEMI upon admission, her age of 32 years old was younger than the general presentation age of SCAD. 

Management of the condition can vary based on patient presentation, and clinical severity, where patients who have high-risk features such as ongoing ischemia, recurrent chest pains, left main artery dissection, ventricular arrhythmias, or hemodynamic instability are treated with revascularization strategies versus conservative management [10, 11]. Our patient was found to have several of the aforementioned features, initially presenting with ongoing chest pain for about seven hours prior to admission and subsequent catheterization. Upon catheterization, coronary angiogram demonstrated dissection of the left main coronary artery extending into the left anterior descending artery, ramus intermedius, and left circumflex artery (LCX) with thrombosis, myocardial infarction (TIMI) 0 flow. The left main coronary artery is less commonly affected in SCAD, making up only 2% of affected cases in some reports, while comprising 13% of the cases in a series that comprised of only STEMI SCAD patients, while the left anterior descending coronary artery (LAD) is often cited as being the most frequently affected vessel, comprising about 40-70% of SCAD cases [6-8, 12]. In addition to having multivessel involvement, this patient also developed ventricular fibrillation and cardiogenic shock, which required the support of vasopressors and the placement of an Impella, and necessitated emergent coronary artery bypass graft (CABG), extracorporeal membrane oxygenation (ECMO), and subsequent heart transplant. 

While coronary angiography remains the gold standard for SCAD diagnosis, management protocols differ based on the predicted severity of the condition. Conservative medical management is generally preferred versus revascularization due to revascularization therapies associated with relatively high rates of failure [7, 11]. In cases where revascularization is necessary, percutaneous coronary intervention (PCI) is preferred over CABG, with CABG serving as a "bail-out" option if the anatomy is not compatible with PCI [11]. In a study comprising of 53 STEMI-SCAD patients, only one patient presented with revascularization failure after the PCI attempt due to propagation of a hematoma extending from the left main coronary artery into the LAD and LCX with associated perforation of the LCX, very similar to the case of our patient. In the same study, only one patient had a PCI stent attempt that had to be followed by CABG, and cardiac transplantation had to be performed in two patients that demonstrated heart failure post-infarction [12, 13]. 

After SCAD, emergent CABG is usually done for severe cases, sometimes requiring mechanical assist devices as a bridge to heart transplantation [9]. While there have been a few cases of patients requiring ECMO, ventricular assist devices, and/or heart transplantation as a result of SCAD, there have been very few cases reported where a patient as young as the one we are reporting had complications that required all the mentioned interventions [7, 14-18]. Additionally, in several of the case reports, the patients that had more severe outcomes requiring ECMO and/or cardiac transplants were either peri/postpartum or had underlying fibromuscular dysplasia (FMD), factors which have been found to be associated with dissection [6, 14-18]. Our patient had her child over 2.5 years ago and was not taking any hormonal therapy, another factor that has been linked with SCAD [6]. Additionally, it has been found that FMD has been associated with coronary artery tortuosity, which has been found to be linked to SCAD [2, 18, 19]. Coronary artery tortuosity can be seen in coronary angiograms and has been characterized by ≥3 consecutive curvatures of 90 to 180 degrees seen at end-diastole in a major epicardial coronary artery ≥2 mm in diameter [18]. However, our presented case did not show the above-mentioned vessel tortuosity. Additionally, this patient's past medical history only consists of a history of asthma, which is significantly different from the conditions associated with SCAD, including connective tissue disorders such as Marfan and Ehlers-Danlos, and systemic inflammatory conditions such as systemic lupus erythematosus, Crohn’s disease, ulcerative colitis, rheumatoid arthritis, and celiac disease [3]. 

Based on this patient's young age, lack of commonly associated conditions predisposing her to SCAD, and severity regarding presentation and outcome, we believe this is a case worth reporting. SCAD often remains misunderstood and underdiagnosed, and with the greater number of atypical cases that are reported, such as this woman without commonly associated risk factors, we take one step closer towards understanding this condition and improving the prognosis of those affected by it. 

## Conclusions

Spontaneous coronary artery dissection is a coronary artery disease that is characterized by intramural hematoma formation with or without an intimal tear. It is not related to atherosclerotic, traumatic, or iatrogenic causes. Although the mean age of SCAD presentation is reported to be 43 to 52 years old, we encourage the clinicians to consider this differential diagnosis in young females who present with typical chest pain with EKG findings suspicious for myocardial infarction. Despite the high success rate of conservative medical management of SCAD with complete resolution of the dissection within a month, careful examination and diagnostic coronary catheterization in high-risk features predict the need for emergent CABG and early aggressive interventions. Further studies on the etiology and associated comorbidities are required to target the primary prevention strategies and mitigate the consequences of this disease.
